# fMRI Investigation of Semantic Lexical Processing in Healthy Control and Alzheimer’s Disease Subjects Using Naming Task: A Preliminary Study

**DOI:** 10.3390/brainsci11060718

**Published:** 2021-05-28

**Authors:** Yen-Ting Chen, Chun-Ju Hou, Natan Derek, Min-Wei Huang

**Affiliations:** 1 Department of Electrical Engineering, Southern Taiwan University of Science and Technology, Tainan 701, Taiwan; ytchen@stust.edu.tw (Y.-T.C.); cjhou@stust.edu.tw (C.-J.H.); da62b206@stust.edu.tw (N.D.); 2 MOST AI Biomedical Research Center at NCKU, Tainan 701, Taiwan; 3 Department of Psychiatry, Chiayi Bran Taichung Veterans General Hospital, Chiayi City 600, Taiwan; 4 Department of Physical Therapy and Graduate Institute of Rehabilitation Science, China Medical University, Taichung City 406040, Taiwan

**Keywords:** Alzheimer’s disease, semantic–lexical processing, fMRI analysis, naming task

## Abstract

For decades, scientists have been trying to solve the problem of dementia, with no cure currently available. Semantic–lexical impairment is well established as the early critical sign of dementia, although there are still gaps in knowledge that must be investigated. In this study, we used fMRI to observe the neural activity of 31 subjects, including 16 HC (Healthy Control) and 15 AD (Alzheimer’s Disease), who participated in the naming task. The neuropsychology profile of HC (Healthy Control) and AD (Alzheimer’s Disease) are discussed in this study. The involvement of FG (Fusiform Gyrus) and IFG (Inferior Frontal Gyrus) shows dominant activation in both of the groups. We observed a decrease in neural activity in the AD group, resulting in semantic deficit problems in this preliminary study. Furthermore, ROI analysis was performed and revealed both hyperactivation and hypoactivation in the AD group. The compensatory mechanism demonstrated during the task, due to the effort required to identify an animal’s name, represents the character profile of AD.

## 1. Introduction

For many years, there have been attempts to cure dementia concerning all different research areas. It was difficult for doctors or professionals to even detect the signs of Alzheimers disease (AD) 45 years ago [1]. Today, the most relevant approach is to detect a deterioration pattern at an early stage or through mitigating preliminary symptoms. Studies of AD are mostly related to semantic memory since it is a common feature of the disease [2,3]. Dementia is a disease that is accompanied by a deterioration in the cognitive function of the elderly. It affects memory, language, thinking, orientation, comprehension, calculation, learning capacity, and judgment [4]. In our previous study, we investigated the physiological responses of Healthy Control (HC) and AD patients by playing games that we had developed to evaluate reaction time and accuracy [5]. In the present study, we applied magnetic resonance imaging (MRI)/functional MRI (fMRI) techniques to observe the neural activity among HC and AD subjects using a naming task that we designed to extract semantic–lexical processing.

Numerous studies working on MRI/fMRI techniques have revealed the neural activity of dementia with various neuropsychology experimental tasks [6,7,8]. Most works investigate episodic memories, as reviewed in [6], and their main goal is to investigate the activation of brain regions corresponding to certain neuropsychological tests related to daily events [6,9]. In common AD neuropathology, the lateral temporal regions are usually affected first, such as the hippocampus and the entorhinal cortex, before broadening to posterior association areas such as the parietal and frontal lobe as the disease progresses [10].

While episodic memory loss is the most common syndrome in AD, language impairment is a common problem and an early sign of AD; naming and fluency deficits are particularly prevalent [11,12,13,14,15]. In the early stage of AD, word retrieval, and semantic- and episodic-memory impairment have been identified [16]. Semantic memory is the general knowledge acquired through the experience of living, consisting of actions, objects, facts, people, relations, and culture [7,8,15,17]. Semantic memory is critical for investigation since it is related to general facts and knowledge, and could affect the daily activities of patients and their personal relationship with people around them (e.g., their family). While much research [7,14,18,19,20,21,22] strongly supports that AD patients have impairment in semantic processing, there are still many knowledge gaps that require further study to fill. The nature is still actively debated particularly concerning where the semantic impairment was caused by the loss of specific knowledge or impairment of access to the knowledge itself [11,19] Therefore, the scope of this research involves observing the semantic–lexical processing utilized by the naming task among AD and HC subjects.

Naming and verbal fluency tasks are commonly used to assess language dysfunction in AD as they require intentional processing and can be a marker of semantic memory impairment [7,23,24]. Previous research has implied a deterioration in semantic knowledge rather than just simple impaired retrieval ability [11,24]. Along with this, patients tend to make persistent mistakes with the same object. In other words, semantic knowledge may be disintegrated [12]. The word retrieval process includes two attributes: Accessing words (lexical processing), and the meaning of the words (semantic processing). In AD, these two processes can be impaired, which leads to poor task performance [25].

To the authors knowledge, there are few studies using fMRI to investigate neural activity in AD related to language processing, specifically using naming tasks to utilize semantic processing, and there is still considerable divergence between them [11,26]. Wierannga et al. [11,25,27] performed an investigation within this scope, and their focus was on the feature–category processing of naming tasks to evaluate the category of living vs. non-living things using three categories (tools, animals, and vehicles) in their latest study [11]. In their results, they found that the responses of AD participants were slower compared to those of HC participants in all three categories, but responses for the animal category were more accurate than responses for vehicles and tools [11]. Paek [26], in their earlier test–retest reliability study, used noun and verb confrontation naming for their task. They focused on a comparison between tasks during intervals of one week and two months. They suggested that more than one fMRI scan is necessary in order to have the authentic pattern of neural activation, particularly for AD subjects. However, this is a key issue for MRI/fMRI acquisition data since it is hard to maintain the administration of a longitudinal study, and handling AD subjects to follow up the task during the scanning process is not easily achieved [28]. Later, in their pilot study [29], they investigated the neural correlates of verb fluency performance and used one-time scanning only among subjects.

In this work, we specifically designed the naming task to target semantic–lexical processing related to the neural activity of HC and AD subjects. Naming tasks are considered as cognitive tasks that can be used as a measurement tool of semantic impairment for AD [30]. A task must be designed as simply as possible due to the condition and limitations of the elderly, but also to be as effective as possible to stimulate brain activity during the fMRI scanning process. We used naming tasks of colored animal pictures and compared them to fixated-cross images as the baseline to obtain the contrast of neural activity. The subjects were instructed to silently name or to just think the name of the animal while they were being scanned.

We hypothesized that there was semantic–lexical impairment in the pattern of neural activation among AD subjects compared to that of the HC group that could be exposed by this task. Brain regions such as the temporal, frontal, and parietal left perisylvian regions (the language region) could make a more significant contribution to the development of networks supportive of language processing components inclusive of recognition, syntax, and semantics [31].The regions in the anterior temporal lobe and frontal lobe, which are believed to be the semantic network regions, were investigated in this study. Extensive research revealed that the anterior temporal lobe (ATL) functions as a semantic hub that is primarily responsible for semantic knowledge [16,32,33], particularly in the fusiform gyrus [11,25,32,34,35]. The involvement of the frontal lobe in the process of the lexical retrieval of a word is within the inferior frontal gyrus [16,25,32,34,36]. The decades-old question still remains: Is a semantic deficit caused by semantic knowledge deterioration, lexical-retrieval impairment, or both. These processes are rarely separated [2,3,11,16,20,33]. Impaired access to the ATL could be interpreted as a deficit of semantic knowledge, while inferior frontal gyrus (IFG) deterioration of the frontal lobe is related to cognitive deficit, particularly in the retrieval process of words.

## 2. Materials and Methods

### 2.1. Participants

The study protocol was approved by the Institutional Review Board (or Ethics Committee) of Taichung Veteran General Hospital. A total of 31 elderly individuals participated in this preliminary study. Sixteen healthy controls (aged in the range of 56–77 years old; 11 females and 5 males) and 15 Alzheimers disease patients (aged in the range of 72–87 years old; 11 females and 4 males) as categorized by the Montreal Cognitive Assessment (MoCA) score and diagnosed by the clinician. One of the HC subjects (s2) was excluded because the T1 image was shifted during the scan, and one of the AD subjects (s23) was excluded due to the health condition of the patient (not being able to cooperate with the clinical evaluation). Participants with dementia were diagnosed by clinicians (the doctor and the psychologist expert in this particular area). All participants went through neuropsychology testing (MoCA) to determine their cognitive impairment level. All participants were confirmed to have no visual impairment and could clearly see the animal pictures on the mirror projected in the fMRI room. All participants were Taiwanese, meaning they could name the animal in their native language, which was Chinese or Taiwanese. All participants were right-handed. See Table 1 for more details of the subject demogragraphics.

### 2.2. Protocols and Materials

**Figure 1:**
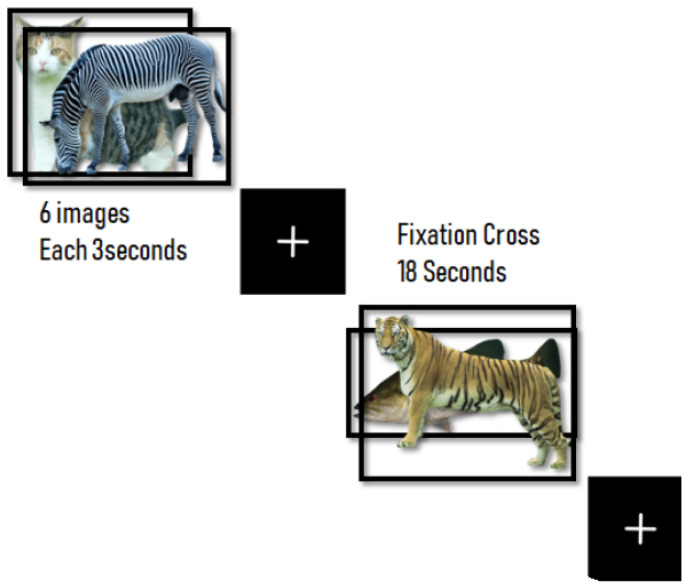
The naming task designed with Unity.

The task for the stimulation was designed with 10 s of instructions (i.e., Please think of the name of the animal) in Chinese, 18 s of a block-design naming task (a total of 8 blocks), and 18 s of cross-fixation for the baseline (a total of 8 blocks). Each block of naming tasks contained 6 pictures that were displayed for 3 seconds per image (shown in Figure 1). Before the fMRI scanning process, all participants practiced the task on a personal computer (PC) and were instructed to think of the name of the animal instead of speaking it while in the fMRI room and to rest (concentrated on the cross) during the fixation task. During the scanning process, participants were instructed not to move their head to avoid movement artifacts. After the fMRI scanning process, the participants were asked the name of the animal while having a discussion with the clinician to make sure that they followed all the procedures and to know if they had any problems during the scanning process. The software was designed with UNITY game development and C sharp as the programming language. To synchronize the fMRI scanning software with the stimulation on the other computer, we used E-Prime software with a serial USB port connected to Arduino (our design connector). This can synchronously trigger the stimuli with the MRI machine. The stimulus computer was then connected to the projector outside of the MRI machine room, and the mirror was placed above the head coil to reflect the projection of the stimuli. We chose 48 images of familiar animal pictures after discussing with the psychologist and doctors to make sure they were familiar animals for Taiwanese individuals. Images were acquired from [37].

### 2.3. FMRI Data Acquisition, Preprocessing, and Statistical Analysis

MRI images were acquired using a 1.5 T Signa HDx system (GE Healthcare, United Kingdom) with a standard RF receiver head coil in Chiayi Branch Taichung Veterans General Hospital, Taiwan. FMRI/echo planar imaging (EPI) angle = 90${}^{\circ }$, TR = 3000 ms, TE = 35 ms, FOV =24 × 24 mm${}^{2}$, 37 interleaved descending slices, voxel size = 3.00 × 3.00 × 3.00 mm${}^{3}$. Image preprocessing and statistical analysis were processed using SPM 12 (Wellcome Trust Centre for Neuroimaging, London, UK, http://www.fil.ion.ucl.ac.uk/spm/l, accessed on 8 January 2020) which was implemented on MATLAB version 2016b. Dicom images were converted into NIFTI files (both T1 and functional images). All preprocessing images (slice timing correction, realignment or motion correction, spatial normalization, coregistration, and smoothing using FWHM: 8 mm) were performed with cautious observation. Statistical analysis was proposed using a general linear model (GLM) to measure each voxel activity. Each subject had two conditions, the naming task and rest (cross -fixation), which were used as regressors in the model. Parameter motions (as the results of the realignment process) were also included as multiple regression to reduce the motion effect during the scanning process. T-contrast was used to calculate the contrast within subjects for each of the conditions to produce the TMAP image. In group analysis, we used one sample t tests within the group (both HC and AD) and two sample t tests for group comparison. Region of interest (ROI) analysis was conducted using SPM to observe the contrast of parameter estimates within each subject, and the Marsbar (http://marsbar.sourceforge.net/about.html, accessed on 8 January 2020) toolbox was used for the percentage of activated voxels within the fusiform gyrus and inferior frontal gyrus (pars triangularis). These regions were extracted on the basis of the anatomical region using the AAL MNI template. The visualization and interpretation of the statistical parameter map images (TMAP) were performed by using the BSPMVIEW tool box (http://www.bobspunt.com/bspmview/, accessed on 8 January 2020).

## 3. Results

### 3.1. Healthy Controls

#### 3.1.1. Whole Brain Analysis

**Figure 2:**
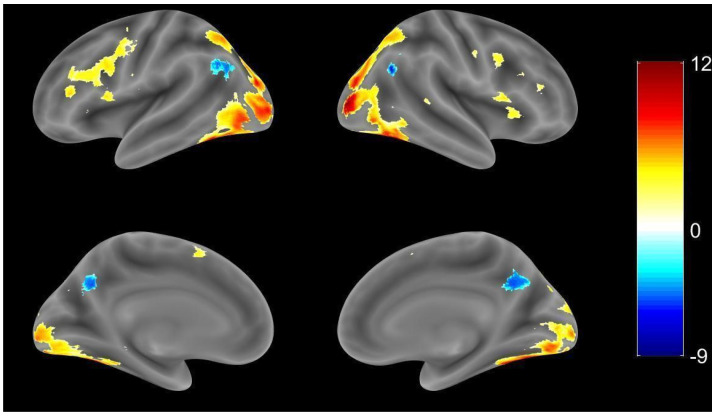
Whole brain activation patterns for the HC group (*p* value < 0.001, uncorrected).

Individual TMAP images were observed one by one to make sure there was a common pattern of semantic task-related regions within each subject. Most participants demonstrated upregulation in the temporal lobe and frontal lobe, as these areas are known as semantic areas, even though the number of activation voxels varied between each subject. One sample t tests were used for group analysis in the HC group. Compared to the cross-fixation (resting period), the HC group showed normal upregulation in the bilateral hemisphere during the naming task, as shown in Figure 2 (threshold at *p* value < 0.001). There was dominant activation, particularly in the fusiform gyrus (FG) bilaterally and the left IFG, and in the pars triangularis, which is believed to be responsible for semantic tasks. However, left and right hemisphere clusters were connected in this threshold value, which makes it important to conduct ROI analysis (see Table 2 for all activations of the brain regions).

#### 3.1.2. Region of Interest (ROI) Analysis

Table 2 shows that the number of voxels (extent) was indistinct in some cases, such as for the left and right fusiform gyrus. When we performed ROI analysis for the percentage of activation voxels within these regions, the left fusiform gyrus was larger than the right fusiform gyrus (L FG = 29.40%, R FG = 28.11%; threshold value > 3.4) and the right inferior frontal gyrus (pars triangularis) was larger than the left inferior frontal gyrus (pars triangularis) (L IFG = 0.42%, R IFG = 0.57%; threshold value > 3.4). This is because the left IFG (pars triangularis) is larger in the AAL template. Furthermore, when we extracted the mean of the contrast using parameter estimates in these regions, among subjects, the right hemisphere of the FG had a higher value than the left FG (R FG =0.836, L FG = 0.693) and was significantly different than the *p* value (0.022, t-stat = −2.5658). The same was true for the right IFG (pars triangularis), which had a higher value than that of the left IFG (pars triangularis) (R IIFG = 0.422, L IFG = 0.374) with no significant differences.

### 3.2. Alzheimers Disease

#### 3.2.1. Whole Brain Analysis

**Figure 3:**
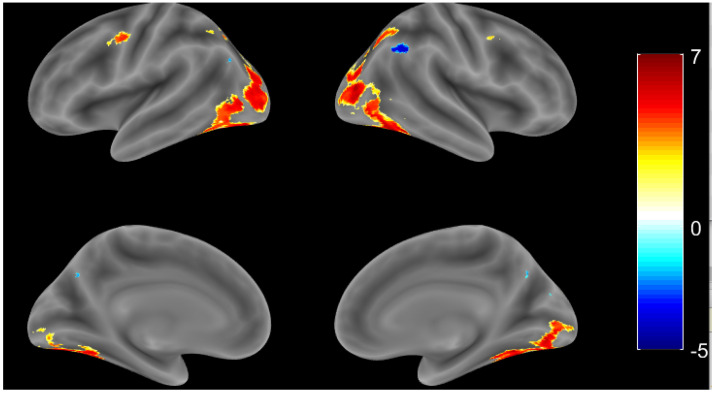
Whole brain activation patterns for the AD group (*p* value < 0.001, uncorrected).

AD patients showed more varied patterns of activation for each individual during the naming task than during the cross-fixation task when individual observations were performed. Single subject analysis was considered necessary for each AD case (see Section 4 for further details). However, the AD group result still showed significant activation of the fusiform gyrus and inferior frontal gyrus in the bilateral hemisphere, even with a smaller number of voxels. See Figure 3 and Table 3 for the activation regions of the AD group with a threshold at *p* < 0.001, uncorrected.

#### 3.2.2. Region of Interest (ROI) Analysis

In contrast to the HC group, the percentage of activated voxels of the AD group in the right fusiform gyrus was slightly larger than that in the left fusiform gyrus (R FG = 22.92%, L FG = 22.34%, threshold value > 3.4), and the left inferior frontal gyrus (pars triangularis) was larger than the right inferior frontal gyrus (L IFG = 0.95%, R IFG = 0.77%, threshold value > 3.4). The contrast estimate of both regions within this group showed that the right hemisphere had a higher mean value than that of the left hemisphere, with no significant difference found (R FG =0.481, L FG =0.466; R IFG = 0.186, L IFG = 0.148).

### 3.3. Group Comparison

#### 3.3.1. Whole Brain Analysis

**Figure 4:**
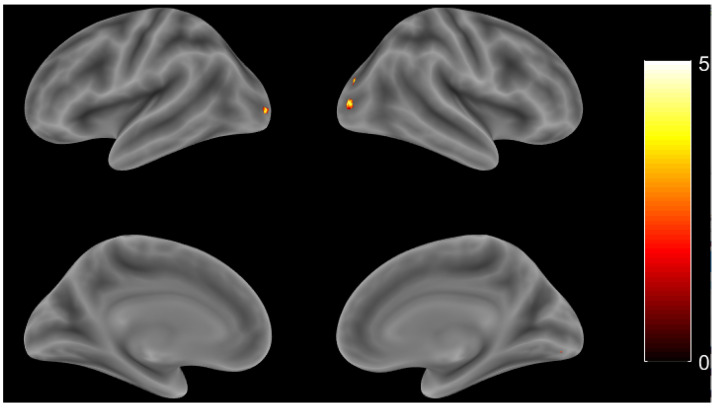
Whole brain activation patterns for HC compared the AD group (*p* value < 0.001, uncorrected).

In the group comparison, two sample *t* tests were performed to observe activation in both groups. When the HC group was compared to the AD group, there was a larger activation area found in the R occipital mid, L inferior occipital gyrus, R cerebellum (VI), and R fusiform gyrus (threshold at *p* value < 0.001, uncorrected). Figure 4 and Table 4 show more details. Unfortunately, no voxel survived after applying the *p* < 0.001 (uncorrected) threshold for the AD group compared to the HC group in this study.

#### 3.3.2. Region of Interest (ROI) Analysis

**Figure 5:**
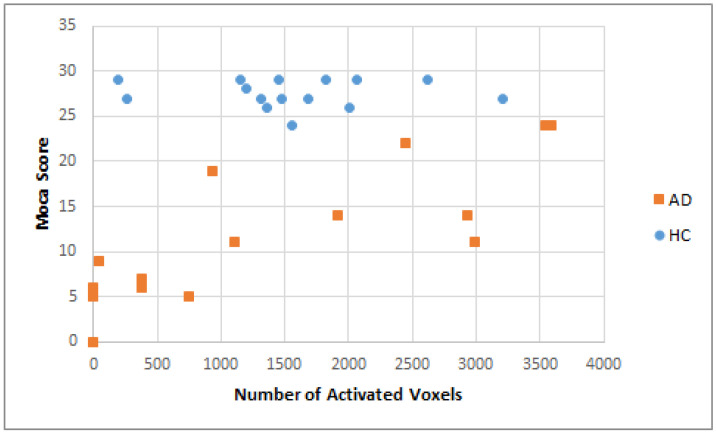
Number of activated voxels related to MoCA score in AD and HC groups (threshold value > 3.4).

Figure 5 shows where the trend of the number of activated voxels among the HC group was centered, while the AD group was more dispersed in the fusiform gyrus and inferior frontal gyrus (pars triangularis). Both hyperaction and hypoactivation were found among individuals with AD. Even though the number of cases was relatively small, this could be representative of the complexity of the neuropathology pattern of AD in general [28].

**Figure 6:**
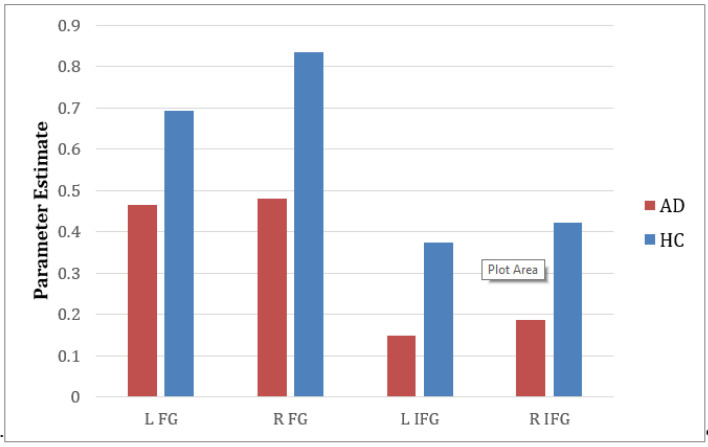
Comparison of parameter estimates between left and right hemispheres. L FG = left fusiform gyrus, R FG = right fusiform gyrus, L IFG = left inferior frontal gyrus, R IFG = right inferior frontal gyrus.

In parameter estimate analysis, we found that the right hemisphere of the fusiform gyrus showed significant differences between these two groups, with *p* value = 0.008 and *t* stat value = 2.840; see Figure 6 for the comparison between the left and right hemispheres of the fusiform gyrus and the inferior frontal gyrus (pars triangularis) between the HC and AD groups.

## 4. Discussion

Several brain regions that are well-known semantic networks, such as the fusiform gyrus, a part of anterior temporal lobe and inferior frontal gyrus (pars triangularis), were located in the frontal cortex and appeared in both the HC and AD groups, as expected in our results.

### 4.1. Healthy Control Group

The overall result indicated that the pattern of activation among individuals in the HC group in these regions was found to be similar, even though there were some clusters that had a lesser amount of activation in some subjects. Similarities could be recognized in these regions among HC individuals who showed normal activation of the semantic network. Our findings indicate that the anterior temporal lobe, particularly in the fusiform gyrus, was more responsible for general linguistic knowledge, the identification and comprehension of facts, and stored concepts. As the number of activated voxels was higher, this corresponds to a greater involvement of the temporal lobe. The involvement of the frontal lobe, specifically the inferior frontal gyrus (pars triangularis), was accountable, and it is related to the lexical and retrieval process of finding a correct word [28]. The fusiform gyrus activation result was high in the HC group, which indicates that the healthy patients were actively engaging in the task [11]. This is in contrast to [38], who proposed significant dominance for the left hemisphere in most individuals of the HC group. Their results denoted that the left hemisphere dominantly played a role in accomplishing developed semantic tasks for HC patients. Interestingly, our study presented greater activation in the right hemisphere, both in the fusiform gyrus and the inferior frontal gyrus.

### 4.2. Alzheimers Disease Group

The domination of activation in ATL, particularly the fusiform gyrus, among AD subjects was found to be similar to that in the HC group in terms of location, but with decreased activation. The evaluated values from the fusiform gyrus were consistently large. The fusiform gyrus was also used in [11] as the region of interest, and it may play a significant role in the activation of semantic memory, such as being able to recognize general knowledge about objects and link words with their associated meanings. Fusiform gyrus activation was greater among the HC group, which signified the ability of healthy subjects to process the meaning of the words being named. The fusiform gyrus in the AD group, on the other hand, showed decreased activation, and thus our preliminary assumption is a semantic–lexical processing deficit. The results from this study coincided with the review explanation in [28] that there was hyperactivation in both the temporal and frontal areas of the brain among healthy subjects, while conversion from hyperactivation to hypoactivation was observed for AD patients. A compensatory mechanism during the retrieval process exists, which showed as hyperactivation when the AD subjects were putting a lot of effort into finding or remembering the correct word. These hyperactivations in different regions, which could be the compensatory mechanisms occurring, are also discussed in [11,29,39]. The simultaneous increased activation of the temporal lobe with the advancement of AD extenuates the patients difficulty or inability to retrieve words [33]. However, some of our AD results expressed decreased activation or even the absence of activation, which could also be a consequence of semantic–lexical processing deficiency. This may represent induced cognitive decline or dysfunction in retrieving and processing of a words meaning. In line with [16], our findings highlight hypoactivation in the temporal area of the brain as a result of cognitive deficit in the semantic–lexical knowledge of AD subjects. Elderly patients diagnosed with AD had a displaced data pattern that resulted from having difficulty with accomplishing a naming task. The inferior frontal gyrus was engaged to a greater extent in AD subjects in comparison with control group subjects during semantic task experiments. Imperative to the understanding of this psychological phenomenon is the contribution of recent studies, which indicate that the involvement of the anterior and ventral frontal brain areas, particularly the IFG, can gauge the capacity of an individual to correctly recognize a particular word [33]. The left inferior frontal gyrus reflected more intense and significant activation in AD patients when they were having a hard time remembering the appropriate word to define the naming task activity at hand. The frontal region of the brain is accountable for retrieval processing ; that is, obtaining the origin or concept of a word. However, AD subjects have a smaller value for the parameter estimates in those regions compared to HC subjects. Saykin et al. [39] postulated that hypoactivation in frontal regions in the left hemisphere of the brain is a direct result of the semantic impairment of Alzheimer’s patients.

### 4.3. Inconsistent Pattern of Brain Activity

A scattered trend of hyperactivation and hypoactivation was observed among AD subjects when we performed the ROI analysis by the number activation of voxels. On the other hand, visual results from the HC group (as demonstrated in the ROI analysis) conveyed a concentrated pattern, which indicated similar or consistent activation. Both hyperactivation and hypoactivation are common phenomena in AD, which make the process of statistical analysis difficult and the pathophysiology of AD hard to define [28]. There is no current method to characterize the pattern of activation in this frame of reference [28,39]. However, individual observation is necessary to explain this, since not every AD patient has the same pattern of activation. Further diagnosis by a clinician related to the diagnosis of the cognitive impairment of AD is necessary.

### 4.4. Other Active Regions

As this naming task required the visual imagery of animal pictures, the activation of the occipital lobe unsurprisingly played a dominant role in the number of voxels that were activated in most subjects. The L precentral gyrus was also found to be activated in [26], which is part of the inferior frontal gyrus, appearing both in HC and AD groups. The posterior medial frontal cortex was also found to be activated in the HC group, which is related to decision making. The activation of the temporal pole that is specific to the animals discussed in [38] also appeared in their HC group. However, an inconsistency occurred, and the number of activated voxels that were relatively small in both the HC and the AD groups were discussed. Further investigation should be conducted related to this.

## 5. Study Limitations and Future Directions

The complexity of abnormal pattern activity in AD makes it difficult to find an appropriate way to chart brain regions. Both hyperactivation and hypoactivation appeared as common patterns in dementia in this study. The problem with the statistical tools that are used currently is that they are limited by the homogeneity or similarity of the mean activation of the population within regions that had no distinct pattern of neuropathology in AD. Individual analysis needs to be conducted, along with an assessment of the clinical background of AD subjects. While there is no current effective way of mapping AD, as described in [28], longitudinal studies along with machine-learning techniques will be the future direction of our work, along with larger samples. Our challenge is to maintain the consistency of the procedure and administration of this study while developing new methods, such as those used in multivoxel pattern analysis (MVPA) and correlated with the MRI analysis. The involvement of artificial intelligence is useful for recognizing activation patterns in the semantic network of AD for further study.

## 6. Conclusions

This study presents one approach to understanding the biological mechanisms of AD in semantic–lexical processing. First, we designed a simple protocol for measuring the neural activity of the semantic–lexical network utilizing the naming task designed specifically for the elderly. This has been demonstrated to be effective, as our target group is also the AD Group. Then, we found two regions with predominating activation that are associated with the semantic–lexical network within the temporal lobe and frontal lobe, respectively, IFG and FG. The involvement of regions in the temporal and frontal lobes, which are believed to be the semantic network, were exposed as expected and observed in subjects in both the HC and AD group in this study. The pattern of the lexical Vsemantic network in AD patients showed abnormal upregulation, as suspected. The number of voxels of the temporal lobe, particularly in the fusiform gyrus, showed diverse results among individuals with AD, which led to abnormal common patterns developing in AD [28]. In line with [16], the frontal lobe, which is responsible for executive function, is not easily to be recognized impaired in the early period of AD. Cognitive function carried by the temporal lobe has more impairment, while the involvement of the IFG does not show an obvious pattern of activation, or might be less affected compared to the FG [16]. In other words, this could lead to only semantic knowledge being impaired among AD subjects, while word-retrieval ability or processing and other related cognitive abilities remain intact. Group analysis within the subjects and between the HC and AD groups was performed. However, because there is a distinctive pattern of activation among dementia subjects, and since the number of cases was relatively small, individual-subject analysis based on their clinical report for future research was considered to scrutinize more issues related to the AD profile. The variability of neural activation in AD patients made it challenging to find the original pattern of the neural pathophysiology of AD within the scope of this research. There appears to be both hyperactivation and hypoactivation, which makes it difficult to analyze using current statistical tools. However, modern techniques such as artificial intelligence and other computational methods could be tools to use in future studies, with more data needed. This preliminary study also opens up access to the further investigation of language deficit, specifically in semantic tasks in AD, and by providing a new database for further studies. In the future, changes in BOLD signals as revealed by fMRI might be useful in evaluating the clinical manifestation of dementia. The further classification of clinical manifestations and sub-items of semantic tests is worth discussing. Additionally, there are currently some studies focusing on the clinical evaluation of serious games for cognitive function as our primary project [5]. The work to assess other cognitive abilities is primarily based on the instruments in MoCA for the elderly, especially those with Alzheimer’s disease. Extended research will be conducted in the future based on these findings.

## Figures and Tables

**Table 1:** Subject demographics of HC (Health Control) and AD (Alzheimer Disease).

Sex	People		Mean and Standard	Mean and Standard
			Deviation of Age	Deviation of Moca
HC			68.33 + 5.47	28.533 + 1.45
	Male	5		
	Female	10		
AD			79.92 + 4.39	13.64 + 6.78
	Male	4		
	Female	10		

**Table 2:** Activation of brain regions for the HC group (*p* value < 0.001, uncorrected).

Region Label	Extent	T Value	MNI Coordinate		
			x	y	z
R fusiform gyrus	11,475	12.7884	42	−60	−14
L fusiform gyrus	11,475	10.5606	−20	−90	−2
L cerebellum (III)	106	5.8934	−4	−48	−14
L IFG (p. triangularis)	1018	5.7927	−38	32	10
L precentral gyrus	1018	4.955	−46	0	48
L posterior-medial frontal	264	5.7539	0	8	62
L IFG (p. orbitalis)	210	5.591	−52	20	−2
R insula lobe	316	5.479	36	20	4
R temporal pole	316	4.8344	58	10	2
R precentral gyrus	278	5.2847	52	8	44

**Table 3:** Activation of brain regions for the AD group (*p* value < 0.001, uncorrected;).

Region Label	Extent	T Value	MNI Coordinate		
			x	y	z
L fusiform gyrus	5421	7.300	−32	−76	−12
R lingual gyrus	5421	7.288	22	−84	−6
R fusiform gyrus	5421	7.237	36	−56	−12
L putamen	24	5.508	−26	−22	4
L precentral gyrus	114	5.110	−52	2	46
R middle frontal gyrus	13	4.520	42	6	46
R thalamus	9	4.497	28	−28	6
L superior parietal lobule	24	4.480	−22	−68	50
L middle occipital gyrus	40	4.468	−24	−60	42
L IFG (p. triangularis)	6	4.284	−52		

**Table 4:** HC and AD group comparison of the activation of brain regions (*p* value < 0.001, uncorrected).

Region Label	Extent	T Value	MNI Coordinate		
			x	y	z
R lingual gyrus	138	5.034	24	V82	6
R cerebellum (crus 1)	70	4.476	42	V64	V24
L inferior occipital gyrus	36	4.311	V24	V92	0
R cerebellum (VI)	42	4.192	24	V78	V18
L cerebellum (VI)	37	4.028	V36	V56	V32
L caudate	10	3.971	V14	V28	26
L cerebellum (VI)	6	3.797	V8	V62	V18
R lingual gyrus	9	3.716	14	V86	V8
L middle occipital gyrus	40	4.468	V24	V60	42
L IFG (p. triangularis)	6	4.284	V52	24	30

## Data Availability

Data available on request due to restrictions eg privacy or ethical. The data presented in this study are available on request from the corresponding author. The data are not publicly available due to [Privacy of the Subjects].
